# The Apgar Score in Foals: Association with Clinical, Behavioral and Reproductive Parameters

**DOI:** 10.3390/ani16162536

**Published:** 2026-08-14

**Authors:** Dania Cingottini, Alessandra Bulgarella, Giulia Sala, Maria Claudia Curadi, Paola Marmorini, Giorgia Troiani, Micaela Sgorbini

**Affiliations:** 1 Department of Veterinary Sciences, University of Pisa, 56124 Pisa, Italy; dania.cingottini@phd.unipi.it (D.C.); maria.claudia.curadi@unipi.it (M.C.C.); g.troiani@studenti.unipi.it (G.T.); micaela.sgorbini@unipi.it (M.S.); 2 Department of Veterinary Medicine and Animal Science, University of Milan, 26900 Lodi, Italy; giulia.sala1@unimi.it; 3 Independent Researcher, 56124 Pisa, Italy; ninnaro@tiscali.it

**Keywords:** foal, Apgar score, clinical parameters, behavioral parameters

## Abstract

The Apgar score is commonly used to assess neonatal vitality in foals, although its clinical relevance remains unclear. This retrospective study evaluated 144 foals investigating associations between a four-parameter Apgar score assessed within 5 min of birth as well as selected neonatal and maternal variables. Inclusion criteria required availability of Apgar scores and body temperature measurements. Maternal, reproductive, and peripartum determinants were evaluated using ordinal logistic regression. Spearman’s rank correlation, Kruskal–Wallis analysis, and Fisher’s exact test were applied as appropriate according to type and distribution. Multiple comparisons were controlled using the Benjamini–Hochberg false-discovery-rate procedure. Most foals showed good neonatal vitality (median Apgar score 8/8, IQR 7–8; 91.7% had scores ≥ 7/8). Higher Apgar scores were associated with higher heart rate, respiratory rate, and shorter time to sternal recumbency, therefore supporting the score’s internal consistency. No associations were found with neonatal behavioral milestones, blood glucose, IgG concentrations, or maternal and reproductive variables. Umbilical abnormalities were not associated with low Apgar score after multiple-comparisons correction. However, few foals scored ≤ 6/8, which limited the evaluation of clinically compromised neonates. Overall, the Apgar score appears to be a practical tool for evaluating immediate neonatal vitality in foals. However, the associations with subsequent neonatal and maternal variables were limited.

## Simple Summary

Assessing the health of newborn foals immediately after birth is key to identifying animals that may require early veterinary attention. The Apgar score, which was first developed for human babies, is commonly used for foals to evaluate their vitality shortly after birth. However, little information is available regarding how this score relates to clinical findings in foals and reproductive factors in mares. In this retrospective study, 144 mare–foal pairs were analyzed to investigate these relationships. Most foals showed a high Apgar score, indicating good neonatal vitality. Foals with higher Apgar scores showed stronger cardiorespiratory function and a shorter time to sternal position, supporting the internal consistency of the score. Overall, no clear relationships were found between the Apgar score and most of the other neonatal or maternal variables. These findings suggest that the Apgar score is a practical tool for assessing a foal’s condition immediately after birth. However, it would seem to be less useful for predicting later clinical findings or early neonatal behaviors, such as standing and nursing.

## 1. Introduction

The transition from intra-uterine to extra-uterine life in the foal depends on a complex and well-coordinated series of physiological adaptations, particularly involving the respiratory and cardiovascular systems [1]. At birth, the foal is physiologically hypoxemic and hypercapnic; these conditions stimulate the first gasping breaths and promote lung inflation [2]. The establishment of effective pulmonary ventilation subsequently triggers the cardiovascular adjustments necessary for successful extrauterine adaptation [1]. Because this transitional phase is particularly delicate, early recognition of abnormalities and prompt intervention are critical to ensure adequate adaptation [1]. In apparently viable foals, subtle disturbances during the early transitional period may rapidly compromise the foal’s clinical status, leading to multisystemic neonatal disorders characterized by nonspecific signs such as weakness, prolonged recumbency, depression, and reduced affinity for the mare [3]. Foals exhibiting inconsistent or absent nursing behavior, progressive weakness, or recumbency should be considered critically ill and require immediate supportive intervention [4]. Although overall neonatal mortality in foals is relatively low, reported rates of up to 1.7% within the first 48 h of life highlight the importance of early monitoring and timely intervention [5,6]. Clinical observations indicate that 70–80% of neonatal cases present within the first 48 h, and among foals that do not survive, approximately 70% die during this initial period [4]. Mortality during this timeframe is often associated with inadequate or delayed neonatal care, as well as metabolic, pulmonary, cardiovascular, and thermoregulatory disturbances occurring immediately after birth [5,6]. Recumbent neonates presented during this critical period require urgent attention, including rapid referral, prompt evaluation of essential organ function, and immediate initiation of targeted supportive therapy to improve survival outcomes [4]. Evaluation of the newborn foal therefore represents the earliest opportunity to assess viability and determine whether immediate intervention is required. This could include oxygen supplementation, positive-pressure ventilation, blood transfusion, or antimicrobial therapy. Risk assessments should consider maternal, parturition, and management-related factors even before the foal is physically examined. While certain high-risk situations (e.g., dystocia or peripartum maternal disease) clearly indicate the need for clinical attention, in many cases the risks may be less evident. Therefore, even apparently normal foaling requires careful monitoring of the foal’s transition and a readiness to intervene when necessary [7]. Consequently, an early complete physical examination and standardized monitoring protocols are fundamental to promptly identify foals at risk and guide timely clinical management [8,9]. Among the tools available, the Apgar score represents a simple, rapid, and widely validated method for evaluating neonatal vitality. The Apgar score was originally developed in human medicine to evaluate the immediate condition of the newborn [10].

In equine neonatology, Apgar scoring should similarly be interpreted primarily as an immediate clinical assessment tool. According to Vaala [3], the 1 min Apgar score mainly reflects the foal’s intrapartum condition and provides indirect information on umbilical blood flow, pH, acidosis and perinatal intrauterine asphyxia. The score thereby helps to identify foals that may require prompt resuscitation or closer early monitoring. By contrast, Apgar scores obtained at 5 and 10 min have been associated with neurological outcomes and with the adequacy of postpartum resuscitation in both human and veterinary medicine [11]. A simplified four-parameter Apgar scoring system, derived from the original human score described by Martens (1982) [12] and Vaala (1999) [13], has been developed and validated for use in equine neonates. This provides a rapid assessment of foal viability based on readily obtainable clinical parameters in both hospital and field settings [12,13,14,15,16,17,18]. In horse foals, The Apgar score has been associated with placental features, blood gas analysis, and hematological and biochemical parameters, thus supporting its value as an indicator of immediate neonatal vitality [2,3,7,11,12,13,14,15,16,17,18,19,20]. Cruz et al. [9] reported that clinically healthy foals undergo marked physiological changes during the first 48 h of life. They emphasized the importance of serial clinical examinations and age- and breed-specific reference intervals, whereas Medica et al. (2018) [16] reported associations between placental characteristics and the Apgar score. In donkeys and mules, Apgar scoring has been related to behavioral milestones, including time to sternal recumbency, standing and nursing, as well as cardiorespiratory and laboratory parameters, highlighting species-specific differences in neonatal adaptation [5,21]. Similarly, Fonte and colleagues [22] reported no significant differences in Apgar scores or early neonatal behavioral parameters between foals conceived using conventional embryo transfer and intracytoplasmic sperm injection, thus indicating comparable immediate neonatal adaptation. Despite these advances, previous studies have primarily investigated either clinical and laboratory variables, behavioral adaptation, species-specific reference values or reproductive technologies [5,9,19,20,23]. Placental characteristics have been associated with Apgar scores in horse foals [19]. However, maternal reproductive variables, including gestational length, placental weight, placental evaluation and time to placental expulsion, have rarely been investigated together with detailed neonatal behavioral parameters within the same population. Reproductive variables were investigated because they reflect the quality of the maternal–fetal environment and may influence neonatal vitality, as assessed by the Apgar score.

In addition, because heart rate, respiratory rate and time to sternal recumbency are either components of, or closely related to, the simplified Apgar score, any association between these variables and the score should be interpreted cautiously to prevent circular reasoning. The aim of the present retrospective study was to investigate the relationship between a simplified four-parameter Apgar score and selected mare reproductive variables, together with early clinical and behavioral parameters of newborn foals managed under routine clinical conditions. By integrating maternal reproductive history with neonatal clinical findings and behavioral adaptation within a single cohort, this study addresses an aspect that has not been comprehensively evaluated in previous equine studies.

## 2. Materials and Methods

In this retrospective study, clinical records of a cohort of foals and their mares collected between 2014 and 2024 from a single breeding farm (La Piaggia Srl, Staffoli, Pisa, Italy) were retrieved from the Veterinary Teaching Hospital (VTH, Department of Veterinary Sciences, University of Pisa) database. According to institutional and national regulations, formal ethical approval was not required. Owners’ written informed consent regarding the use of clinical data for research purposes was obtained.

The inclusion criteria consisted of the availability of a 4-parameter Apgar score performed within the first 5 min after birth (Table A1) [7,12,24] and body temperature (BT) measurements [2].

Additional parameters were assessed. The parameters included for foals were: (1) sex; (2) behavioral parameters (Table 1); (3) umbilical remnant (normal/not normal); (4) meconium passage (recorded as yes/no) (either occurring spontaneously or after the administration of an osmotic enema); (5) blood glucose concentration; and (6) serum IgG concentration. Behavioral parameters were visually evaluated from outside the box. Suckling reflex was evaluated at 20 min by inserting a finger into the foal’s mouth and was classified as present, absent or weak [21]. Blood glucose concentration was assessed within 5 min of birth on whole blood, using a point-of-care (POC) analyzer (Pic Gluco Test, Pic Solution, SD Biosensor, Inc., Suwon-si, Republic of Korea). Serum IgG concentration was assessed at 24 h using a semiquantitative ELISA kit (SNAP Foal IgG test, IDEXX Laboratories, Westbrook, ME, USA).

The parameters included for mares were: (1) gestational length; (2) time to placental expulsion; (3) macroscopic placental evaluation [23]; and (4) placental weight.

### Statistical Analysis

Statistical analyses were performed using IBM SPSS Statistics, version 30.0 (IBM Corp., Armonk, NY, USA). Data distribution was assessed using the Shapiro–Wilk test. As continuous variables were not normally distributed, they are presented as median and interquartile range (IQR; 25th–75th percentile). Categorical variables are reported as frequencies and percentages.

Analyses were conducted using available cases, and no missing values were imputed. The number of available and missing observations was reported for each variable.

To evaluate the potential determinants of neonatal vitality, the Apgar score was classified into three ordered categories: ≤6/8, 7/8, and 8/8. A second dichotomous classification, ≤6/8 versus 7–8/8, was used for exact analyses involving sparse categorical data.

Potential maternal, reproductive, and peripartum determinants of the Apgar category were investigated using separate univariable proportional-odds ordinal logistic regression models. The Apgar category was entered as the dependent variable, whereas sex, gestational length, and placental weight were entered individually as independent variables. Sex was included as a categorical factor, while gestational length and placental weight were included as continuous covariates. The proportional-odds assumption was assessed using the test of parallel lines.

Heart rate (HR), respiratory rate (RR), and time to sternal recumbency were analyzed separately as indicators of internal consistency because they are directly included in, or closely overlap with, the components used to calculate the total Apgar score. Their associations with the ordinal Apgar score were assessed using Spearman’s rank correlation.

Associations between the ordinal Apgar score and continuous neonatal variables were also evaluated using Spearman’s rank correlation. For each correlation, Spearman’s rho, the number of observations available, and a bias-corrected and accelerated 95% bootstrap confidence interval based on 5000 resamples were reported.

As a complementary grouped analysis, the distributions of continuous neonatal variables were compared across the three Apgar categories (≤6/8, 7/8, and 8/8) using the Kruskal–Wallis test. Pairwise comparisons were interpreted only when the overall test was statistically significant, and the corresponding *p*-values were adjusted using the Bonferroni procedure.

Because several categorical variables had low event counts and sparse cells, categorical outcomes were analyzed using Fisher’s exact test and the dichotomous Apgar classification. The suckling reflex was classified as normal versus altered (weak or absent), passive transfer of immunity as adequate versus partial or complete failure of passive transfer (FPT), umbilical status as normal versus abnormal, and meconium passage as normal versus abnormal. Odds ratios and 95% confidence intervals were reported when estimable.

To account for multiple testing, the Benjamini–Hochberg false-discovery-rate procedure was applied separately within three prespecified analytical domains: potential maternal, reproductive, and peripartum determinants of the Apgar category; internal-consistency variables; and concurrent or subsequent neonatal variables. Both unadjusted and false-discovery-rate-adjusted *p*-values were reported. Statistical inference was based primarily on an adjusted *p*-value < 0.05.

To evaluate the potential effect of repeated foaling events from the same mare, sensitivity analyses accounting for within-mare clustering were performed. Proportional-odds ordinal regression models were repeated using ordinal generalized estimating equations, with mare identity specified as the clustering variable, an exchangeable working correlation structure, and robust standard errors. Associations involving continuous variables were reassessed using rank-based regression models with cluster-robust standard errors. The sensitivity analyses were used to determine whether accounting for within-mare dependence materially changed the effect estimates or statistical conclusions.

## 3. Results

A total of 186 clinical records collected between 2014 and 2024 were retrieved from the VTH database. According to the inclusion criteria, 42/186 cases (22.6%) were excluded, and 144/186 foals (77.4%) were included in the statistical analysis. Of the excluded cases, 40/42 were the result of a missing Apgar score, whereas 2/42 had missing body temperature measurements.

All mares were Standardbreds and multiparous; data regarding age were unavailable. Overall, 99 mares each contributed a single foal to the study, whereas 21 mares contributed two foals each, and one mare contributed three foals across multiple foaling seasons. All foalings were spontaneous, eutocic and assisted. Information regarding the duration of the stages of labor was unavailable. All foalings occurred at the breeding farm in individual straw-bedded stalls (5 × 5 m) equipped with external windows and video surveillance. None of the foals required resuscitation or oxygen supplementation after birth. Bottle-feeding assistance was recorded in three foals, using colostrum collected from their respective dams. None of the foals received colostrum supplementation. All foals underwent umbilical disinfection with iodine and received hyperimmune tetanus serum as part of the farm’s standard neonatal management protocol. None of the foals received antimicrobial treatment at birth or during the first 24 h of life. Information regarding antimicrobial treatment beyond 24 h was not available.

Sex was available for 141/144 foals (97.9%); 66/141 were females (46.8%), and 75/141 were males (53.2%).

The median Apgar score was 8/8 (IQR 7.0–8.0) and ranged from 3/8 to 8/8. One out of 144 foals (0.7%) scored 3/8, 4/8 and 5/8, respectively, 9/144 foals (6.3%) scored 6/8, 41/144 foals (28.5%) scored 7/8, and 91/144 foals (63.2%) scored 8/8.

The median HR at birth was 84 bpm (IQR 69–109 bpm), the median RR was 60 bpm (IQR 48–68 bpm), and the median BT was 37.5 °C (IQR 37.2–37.8 °C). Compared with the reference ranges, 1/144 foals (0.7%) had an HR below the range, 139/144 had an HR within the range and 4/144 foals (2.8%) had an HR above the range (reference range HR 60–150 bpm) [2]. Compared with the reference ranges, 10/144 foals (6.9%) had an RR below the range, 128/144 foals (88.8%) had an RR within the reference range, and 6/144 foals (4.2%) had an RR above the range (reference range RR 40–80 bpm) [7,11]. Sixteen out of 144 foals (11.1%) had a body temperature measurement below the reference range, 128/144 had measurements within the range and none of the foals had measurements above it (reference range BT 37–39 °C) [2]. The head-raising reflex was recorded in 144/144 foals (100%), with a median time of 1 min (IQR 1–1 min). In 137/144 foals, the reflex occurred within 2 min, while in 7/144 foals (4.9%) it occurred after 2 min.

The suckling reflex was recorded in 81/144 foals (56.3%). The suckling reflex was evaluated at 20 min, and 79/81 foals showed a normal suckling reflex. In 1/81 foals (1.2%), the suckling reflex was absent, and 1/81 foals (1.2%) had a weak suckling reflex.

Time to sternal recumbency was recorded in 142/144 foals (98.6%), with a median time of 3 min (IQR 2–5 min). Of these, 118/142 foals (83.1%) assumed sternal recumbency within 5 min and 24/142 foals needed more than 5 min.

Time to standing was recorded in 144/144 foals (100%) and a median time of 78 min (IQR 60–120 min) was shown; particularly, 113/144 foals stood up within the reference range, and 31/144 foals (21.5%) required more than 120 min.

First suckling time was recorded in 127/144 foals (88.2%) with a median of 140 min (IQR 100–200 min); of these, 93/127 foals (73.2%) suckled within 180 min, while 34/127 (26.8%) required more than 180 min.

Umbilical cord evaluation was recorded in 144/144 foals (100%): the umbilicus was considered normal in 136/144 foals (94.4%), while abnormalities were detected in 8/144 foals (5.6%). Of these, one foal had a twisted umbilical cord, one had a mildly edematous umbilical cord, and for the remaining 6/8 foals, only the presence of an umbilical abnormality was recorded, without further details reported in clinical records.

Meconium passage was recorded in 85/144 foals (59%), while no information was available for 59/144 foals (41%). Enema administration was documented in 54/85 foals (63.5%), explicitly reported as not performed in 9/85 foals (10.6%), and not reported in 22/85 foals (25.9%). Due to the substantial proportion of missing data, statistical analysis was not performed for the enema administration.

Blood glucose concentration was measured in 102/144 foals (70.8%) with a median value of 65.5 mg/dL (IQR 47.8–83.0 mg/dL). Seventy-three out of 102 foals showed a glucose concentration at birth that was within the physiological range (50–130 mg/dL) [7,25,26,27], while 2/102 foals (2.0%) showed hyperglycemia, and 27/102 foals (26.5%) showed hypoglycemia.

Serum immunoglobulin G concentration was assessed in 92/144 foals (63.9%). Of these, 80/92 foals (87%) showed IgG concentrations > 800 mg/dL, indicating a complete transfer of IgG, while 5/92 foals (5.4%) were affected by partial failure of passive transfer (partial FPT) (400 and 800 mg/dL), and 7/92 foals (7.6%) showed complete FPT (<400 mg/dL) [12].

Gestational length was assessed in 96/144 mares (66.7%), with a median value of 338 days (IQR 332–348). Gestational length was <320 days in 4/96 mares (4.2%), >360 days in 5/96 mares (5.2%), and within physiological ranges (physiological range 320–360 days) [25] in 87/96 mares (90.6%).

Time to placental expulsion was recorded in 35/144 mares (24.3%), with a median time of 65 min (IQR 30–146.3 min). Of these, 28/35 (80%) expelled the placenta within physiological timing (3 h) [28], while 7/35 (20%) required more than 3 h. Among the seven cases of retained placenta, 1/7 (14.3%) required oxytocin administration, while 6/7 (85.7%) received no pharmacological treatment.

Placental evaluation was performed in 141/144 mares, with no macroscopic abnormalities observed in 138/141 cases (97.9%). Most retained placentas (6/7, 85.7%) appeared macroscopically normal, whereas 1/7 (14.3%) was edematous.

Placental weight was recorded in 122/144 mares, with a median value of 5.0 kg (IQR 4.2–5.5 kg). Of these, 36/122 (29.5%) had a placental weight below the reference range, 3/122 (2.5%) above, and 83/122 (68%) within the reference values (4.4–7.7 kg) [29].

Table 2 reports the inferential analyses of potential determinants of the Apgar category, internal consistency, and associations with neonatal variables.

As indicators of internal consistency (Table 2), the ordinal Apgar score was positively correlated with HR (Spearman’s ρ = 0.192, BCa 95% CI 0.017–0.357; n = 144; *p* = 0.021) and RR (ρ = 0.511, BCa 95% CI 0.366–0.637; n = 144; *p* < 0.001) and negatively correlated with time to sternal recumbency (ρ = −0.266, BCa 95% CI −0.430 to −0.087; n = 142; *p* = 0.001). These associations remained statistically significant after false-discovery-rate adjustment.

A weak negative correlation was observed between the Apgar score and time to head raising (ρ = −0.203, BCa 95% CI −0.370 to −0.025; n = 143; *p* = 0.015). The Kruskal–Wallis test also indicated a difference among the three Apgar categories (*p* = 0.007). However, pairwise comparisons showed a significant difference only between foals scoring 7/8 and 8/8 (Bonferroni-adjusted *p* = 0.005), with no significant differences involving foals with an Apgar score ≤ 6. Moreover, the correlation did not remain statistically significant after false-discovery-rate adjustment (adjusted *p* = 0.120).

Umbilical abnormalities were recorded in 3/12 foals (25.0%) with an Apgar score ≤ 6 and in 5/132 foals (3.8%) with an Apgar score of 7–8. Fisher’s exact test indicated higher odds of an Apgar score ≤ 6 in foals with an abnormal umbilical finding (OR = 8.22, 95% CI 1.10–50.95; *p* = 0.020). However, this association did not remain statistically significant after false-discovery-rate adjustment (adjusted *p* = 0.080).

Sensitivity analyses accounting for within-mare clustering yielded effect estimates and statistical conclusions consistent with those of the primary analyses. In particular, sex, gestational length, and placental weight remained unrelated to Apgar category, whereas the associations of Apgar score with HR, RR, and time to sternal recumbency remained statistically significant.

## 4. Discussion

The Apgar score is a simple clinical assessment tool to evaluate neonatal vitality in foals [2,3,5,7,11,12,17,20]. However, the relationships between Apgar scores and reproductive, clinical, and behavioral parameters in equine neonates remain poorly explored. This retrospective study therefore evaluated foal viability at birth using a four-parameter Apgar score and investigated its associations with these variables. The Apgar score was used primarily as an immediate clinical assessment tool to evaluate neonatal adaptation at birth, rather than as a standalone predictor of subsequent neonatal outcome. The rationale for using the simplified four-parameter Apgar system, using a reduced number of variables, aimed to minimize observer-dependent variability, reduce the time required for assessment, and improve consistency among veterinarians and breeders with different levels of neonatal experience. This simplification is particularly advantageous on stud farms and in ambulatory equine practice, where prompt recognition of compromised foals is necessary to guide timely resuscitation, referral, or intensive neonatal care.

The significant associations between the Apgar score and HR, RR, and time to sternal recumbency support the internal consistency of the scoring system. Foals with higher Apgar scores showed better cardiorespiratory function and reached sternal recumbency earlier. This confirmed the ability of the Apgar score to reflect the degree of neonatal adaptation immediately after birth [7,11,19,24,30,31,32]. A small proportion of foals exceeded the 5 min threshold for sternal recumbency [21], requiring more than 15 min despite a high Apgar score. This suggests that additional behavioral or maternal factors may influence this milestone beyond systemic vitality.

A weak negative correlation was initially observed between the Apgar score and time to head raising, which suggests that foals with higher Apgar scores tended to raise their heads slightly earlier. However, this association was weak and should be interpreted with caution. Similarly, although an overall difference among Apgar score categories was observed, the only detectable difference was between foals with Apgar scores of 7/8 and 8/8. However, no differences were found between foals with Apgar scores ≤ 6 and the other groups. The lack of differences involving foals with Apgar scores ≤ 6 should be interpreted with caution, as the limited number of foals in this group may have reduced the statistical power. Moreover, the association did not remain significant after false-discovery-rate correction. This suggests that the observed relationship was not sufficiently robust to support a consistent association between the Apgar score and time to head raising.

Umbilical abnormalities were initially associated with an Apgar score ≤ 6/8. However, this association did not remain statistically significant after adjustment for multiple comparisons and should therefore be interpreted with caution. Furthermore, only 12 foals had an Apgar score ≤ 6/8, which limits the statistical power of this analysis. In addition, information on the type of umbilical abnormality was available for only two of the eight foals affected; for the remaining six cases, only the presence of an abnormality was recorded, without specifying its nature. According to the literature, clinical umbilical assessment typically includes evaluation for abnormalities such as heat, swelling, pain, and purulent discharge [32,33,34,35]. However, these specific findings were not documented in the database and therefore could not be retrospectively characterized. Nevertheless, severe umbilical alterations, such as umbilical cord torsion, have been associated with fetal hypoxia and subsequent cardiorespiratory depression, which supports the clinical importance of umbilical assessment in neonatal foals [35]. These findings should thus be considered exploratory. Further studies including a larger number of foals with low Apgar scores are warranted to clarify the relationship between umbilical abnormalities and neonatal vitality.

No associations were identified between Apgar score and suckling reflex, time to standing position, or time to first suckling. Prolongation of these behavioral milestones is generally considered abnormal and may indicate impaired neonatal adaptation [15]. However, the results are consistent with findings in healthy foals (Apgar 7–8/8), in which behavioral milestones generally occur within the expected physiological timeframe regardless of minor differences in Apgar score [5,14,20]. In contrast, significant delays in standing (180 vs. 90 min; normal < 120 min) [2] and first suckling (200 vs. 130 min; normal < 180 min) [17] have been reported specifically in depressed foals with Apgar scores < 6 [14]. Similarly, studies on the Amiata donkey and mule foals found that lower Apgar scores were associated with delayed standing and suckling [5,20], whereas no such association was observed in the present study. This discrepancy may be explained by the generally good physiological condition of the foals included, with 91.7% showing Apgar scores of 7–8/8. This suggests that behavioral delays are primarily associated with severe neonatal depression rather than with minor variations in Apgar score among healthy neonates. Moreover, behavioral adaptation during the first hours of life is inherently variable in equine foals [13,36,37]. The initiation of suckling may be influenced not only by neonatal systemic adaptation, as reflected by the Apgar score, but also by maternal behavior, mare–foal bonding, and the individual temperament of both the mare and foal [21]. It is also possible that weaker foals or those at risk immediately after birth received special treatment, thus improving their ability to achieve later milestones. These factors may therefore have contributed to the observed findings. However, as they were not evaluated in the present study, no further conclusions can be drawn regarding their potential influence. The suckling reflex was evaluated only once, at 20 min after birth, and was classified as present, weak, or absent. The 20 min time point was selected because the physiological onset of the suckling reflex is expected to occur within the first 20 min of life [21]. Foals showing a normal suckling reflex at 20 min were therefore considered to have achieved it within the expected physiological timeframe, whereas those with a weak or absent reflex were considered to have an altered or delayed response. No repeated assessments were performed. Continuous access to the foals during the first 20 min of life was limited due to the management requirements of the breeding farm. The exact timing of suckling reflex onset could not therefore be recorded, and comparisons with studies reporting time-to-onset were not possible.

Only two foals showed an altered suckling reflex at 20 min, while the remaining foals had a normal reflex. Interestingly, one of the two foals with an altered suckling reflex also had a high Apgar score, which suggests that a satisfactory Apgar score does not necessarily predict normal early suckling behavior. However, given the very low number of foals with an altered reflex, no definitive conclusions can be drawn regarding the relationship between Apgar score and suckling reflex. Further studies including a larger number of compromised foals are warranted.

Hypothermia was observed in a low number of foals, whereas most showed rectal temperatures within the physiological range, and no cases of hyperthermia were recorded. The physiological profile of neonates with reduced vitality—reflected by a low Apgar score—has been associated with decreased metabolic activity and impaired thermoregulatory capacity, thus making these foals potentially more vulnerable to hypothermia [38]. Similarly, previous studies on mule and donkey foals reported hypothermia in neonates with a low Apgar score [5,20]. This thus suggests a possible relation between impaired neonatal adaptation and thermoregulatory instability, although statistical correlations were not performed. In the present study, no statistically significant relationship was identified between BT and Apgar scores. This lack of correlation may reflect the low variability in Apgar scoring within our population, where most foals had Apgar scores of 7–8/8. However, as a purely observational finding, the foal with the lowest Apgar score (3/8) exhibited hypothermia (35.5 °C), in line with previous findings in donkeys and mules [5,20,36].

Meconium passage was recorded in just over half of the foals. Among those with data available, most foals showed normal passage, although a substantial proportion received osmotic enema administration. To the best of our knowledge, no studies have clearly established a relationship between Apgar score and meconium passage in foals. No association between meconium passage and Apgar score was identified in the present study. However, this finding should be interpreted with caution because of the substantial amount of missing data and the predominance of healthy foals in the study population. 

In the present study, a correlation between Apgar score and glycemia was not found. Neonatal glycemia reflects the metabolic adaptation to extrauterine life, particularly in compromised foals. However, a consistent linear association with Apgar score has not been established in previous studies [7,13,17,37]. This is also supported by a previous study [16] in which the Apgar score in clinically normal-term foals remained within physiological ranges and showed only weak associations with endocrine and metabolic variables, including glucose, cortisol and adrenocorticotropic hormone (ACTH). The absence of correlation noted in our predominantly vital foals may reflect the narrow physiological range of both the Apgar score (7–8/8) and glycemia, thus limiting the statistical power to detect subtle relationships, as observed in clinically normal foals.

Immunological assessment revealed that most foals achieved IgG concentrations above 800 mg/dL, with only a limited number exhibiting intermediate (400–800 mg/dL) or low (<400 mg/dL) values. No significant association was found between Apgar score and IgG concentration, which suggests that neonatal vitality alone may not adequately be an indicator of passive transfer status. In fact, successful passive transfer is a multifactorial process that depends not only on the foal’s ability to stand and nurse promptly but also on the colostrum quality, the timing and adequacy of colostrum intake, intestinal IgG absorption, and the management interventions implemented during the neonatal period [7,12]. Although severely compromised foals may be at increased risk of FPT because of impaired nursing behavior [13], the Apgar score represents only one component of this complex process. In the present study, 20 foals with serum IgG concentrations > 800 mg/dL required more than 180 min [17] to initiate suckling. This suggests that delayed nursing does not necessarily result in inadequate passive transfer, particularly when adequate colostrum quality, effective intestinal absorption, or timely management interventions compensate for the delay [7,11]. Notably, bottle-feeding with the mare’s own colostrum was provided to three foals, which may have contributed to the adequate passive transfer despite delayed spontaneous nursing. Maternal causes (e.g., low colostrum IgG content < 30 g/L due to mammary leakage, mastitis, primiparity) [7,11] or foal-related issues (e.g., suckling failure from weakness, congenital anomalies, poor gut absorption, genetic immunocompromising disorders) [7] may therefore adversely affect passive transfer independently of neonatal vitality [7,11].

Gestational length fell within the normal range (320–360 days) in most of the mares included. No correlation between gestational length and Apgar score was observed in this study, in contrast with previous reports describing a higher Apgar score in foals born from longer pregnancies [16,18] and better placental quality [18]. This discrepancy, potentially due to gestational length homogeneity within physiological limits, may explain the limited variability within this cohort.

Placental weight was generally within the physiological range, with only a small proportion of mares showing values outside normal limits. No correlation between placental weight and Apgar score was observed, in agreement with previous studies [14,17,36]. The time of placental expulsion was evaluated only in a limited subset of mares. In most of these cases, the placenta was expelled within the expected timeframe, with occasional cases of delayed expulsion or retention reported and a single case requiring pharmacological intervention. No significant association was found between Apgar score and placental expulsion or retention. However, given the small number of mares with available data for this parameter, these findings should be interpreted with caution, and no definitive conclusions can be drawn regarding the relationship between time of placental expulsion and neonatal vitality. However, previous studies report no clear associations between time of placental expulsion or retention and neonatal Apgar score [14,36]. The lack of correlation likely reflects how placental weight influences chronic fetal development (nutrient/oxygen supply over gestation) rather than acute neonatal viability assessed by the Apgar score, which captures immediate postnatal cardiorespiratory and neurological adaptation [36]. Similarly, placental expulsion is driven by maternal hormonal factors (oxytocin, prostaglandin F_2_α) and uterine involution, independent of foal vitality in uncomplicated foaling [17]. These findings indicate that while placental parameters remain essential for gestational health assessment, they have limited predictive value for neonatal vitality at birth in physiologically normal pregnancies. Although our results agree with the existing literature, they should therefore be considered supportive rather than conclusive due to the limited sample size.

This study has several limitations. Firstly, the retrospective nature of this study resulted in incomplete data recording and a substantial number of missing values. Although complete Apgar score data were available, as this was an inclusion criterion, several other clinical parameters were incompletely recorded. This precluded comprehensive statistical analyses for some variables and introduced the potential for missing-data bias, thereby reducing the statistical power and robustness of the findings. Secondly, since the cohort of foals was drawn from a single breeding farm over an extended period, the findings may have limited generalizability due to the single-center design. Additionally, Apgar score assessments can be influenced by parturition duration and progression, potentially affecting neonatal behavioral parameters. The sample size was also relatively limited, particularly with respect to foals with a low Apgar score. Most of the foals had an Apgar score of 7–8/8, whereas only 12 foals out of 144 had an Apgar score ≤ 6/8. This marked imbalance in Apgar score distribution may have negatively impacted the statistical power to detect differences involving clinically depressed foals. This may therefore have limited the robustness of the statistical analysis and the generalizability of the findings to populations with a higher prevalence of neonatal compromise. Finally, this study did not evaluate clinically relevant outcome measures, such as neonatal morbidity, survival, hospitalization, or treatment requirements. The prognostic value of the Apgar score for predicting adverse clinical outcomes could therefore not be assessed.

## 5. Conclusions

In this predominantly healthy cohort, our assessment of the four-parameter Apgar score within the first 5 min of birth was mainly associated with immediate cardiorespiratory and postural adaptation. No robust associations were identified with later neonatal behavioral milestones, blood glucose or IgG concentrations, or maternal and reproductive variables. Overall, these findings support the use of the Apgar score as a practical clinical tool for the initial clinical assessment of neonatal foals. However, it should be interpreted as part of a comprehensive neonatal evaluation rather than as an independent predictor of subsequent clinical or physiological outcomes.

## Figures and Tables

**Table 1:** Reference values for neonatal behavioral milestones.

Behavioral Parameter	Physiological Parameter	References
Head raising	1–2 min	[17]
Time to sternal recumbency	<5 min	[21]
Suckling reflex	20 min	[21]
Time to standing position	<120 min	[2]
Time to first suckling	<180 min	[17]

**Table 2:** Inferential analyses of potential determinants of Apgar category, internal consistency, and associations with neonatal variables.

Variable	Statistical Analysis and Outcome	n	Effect Estimate	95% CI	*p*-Value	FDR-Adjusted *p*-Value
Potential antenatal and peripartum determinants						
Sex, female vs. male	Proportional-odds ordinal logistic regression; outcome: Apgar category ≤ 6, 7, or 8	141	β = 0.532; OR = 1.70	OR: 0.85–3.40	0.133	0.266
Gestational length, per additional day	Proportional-odds ordinal logistic regression; outcome: Apgar category ≤ 6, 7, or 8	96	β = −0.008; OR = 0.99	OR: 0.96–1.03	0.668	0.891
Placental weight, per additional kg	Proportional-odds ordinal logistic regression; outcome: Apgar category ≤ 6, 7, or 8	122	β = −0.009; OR = 0.99	OR: 0.76–1.30	0.950	0.950
Abnormal umbilical finding	Fisher’s exact test; outcome: Apgar ≤ 6 vs. 7–8	144	OR = 8.22	1.10–50.95	0.020	0.080
Internal consistency variables						
Heart rate	Spearman correlation with original Apgar score	144	ρ = 0.192	0.017–0.357	0.021	0.021
Respiratory rate	Spearman correlation with original Apgar score	144	ρ = 0.511	0.366–0.637	<0.001	<0.002
Time to sternal recumbency	Spearman correlation with original Apgar score	142	ρ = −0.266	−0.430 to −0.087	0.001	0.002
Concurrent and subsequent neonatal variables						
Time to head raising	Spearman correlation with original Apgar score	143	ρ = −0.203	−0.370 to −0.025	0.015	0.120
Blood glucose concentration	Spearman correlation with original Apgar score	102	ρ = 0.000	−0.204–0.196	0.996	1.000
Body temperature	Spearman correlation with original Apgar score	144	ρ = −0.050	−0.224–0.123	0.552	1.000
Time to standing	Spearman correlation with original Apgar score	144	ρ = −0.121	−0.289–0.049	0.147	0.392
Time to first suckling	Spearman correlation with original Apgar score	127	ρ = −0.039	−0.211–0.134	0.663	1.000
Altered suckling reflex	Fisher’s exact test; altered reflex in Apgar ≤ 6 vs. 7–8	81	OR = 12.85	0.62–813.45	0.056	0.224
Failure of passive transfer	Fisher’s exact test; FPT in Apgar ≤ 6 vs. 7–8	92	OR = 0.57	0.01–4.74	1.000	1.000
Abnormal meconium passage	Fisher’s exact test; abnormal passage in Apgar ≤ 6 vs. 7–8	85	Not estimable	Not estimable	1.000	1.000

β, regression coefficient; CI, confidence interval; FDR, false discovery rate; FPT, failure of passive transfer; OR, odds ratio; ρ, Spearman’s rank correlation coefficient. For ordinal logistic regression, odds ratios refer to the odds of being in a higher Apgar category. The male sex was the reference category. The proportional-odds assumption was satisfied for sex, gestational length, and placental weight. For umbilical status, the OR represents the odds of an Apgar score ≤ 6/8 in foals with an abnormal versus normal umbilical finding. For suckling reflex and passive transfer, ORs represent the odds of the adverse outcome in foals with an Apgar score ≤ 6/8 compared with those scoring 7–8/8. Exact conditional ORs and exact 95% CIs are reported for Fisher’s exact tests. The OR for meconium passage could not be reliably estimated because of a zero cell and only two abnormal observations. Confidence intervals for Spearman’s ρ are BCa 95% bootstrap confidence intervals based on 5000 resamples. FDR adjustment was performed separately within the three prespecified analytical domains using the Benjamini–Hochberg procedure.

## Data Availability

The data presented in this study are available on request from the corresponding author.

## Abbreviations

The following abbreviations are used in this manuscript:

HR	Heart Rate
RR	Respiratory Rate
BT	Body Temperature
VTH	Veterinary Teaching Hospital
IgG	Immunoglobulin G
POC	Point-of-Care
IQR	Interquartile Range
FPT	Failure of Passive Transfer
ACTH	Adrenocorticotropic Hormone

## Appendix A

**Table A1:** Simplified Apgar scoring system for evaluation of foals 1 to 5 min after birth [7,11,21].

Observation	Assigned Value		
	0	1	2
HR	Not detectable	<60 bpm	≥60 bpm
RR and breathing pattern	Not detectable	Slow, irregular	≥40 bpm, regular
Muscle tone	Lateral recumbency, weakness	Lateral recumbency, moderate muscle tone	Sternal recumbency achieved and maintained spontaneously
Responsiveness	No response	Grimacing, weak withdrawal	Coughing and sneezing

Scores range (obtained by summing the individual contributions) from 0/8 to 3/8: severe depression; 4/8–6/8: mild to moderate depression; 7/8–8/8: normal foal.
